# Differential Consequences of Unilateral Nasal Air-Puff Stimulation on Breathing Pattern and Respiratory System Mechanics in Tracheotomized Rats

**Published:** 2013-02

**Authors:** Morteza Bakhshesh, Esfandiar Heidarian, Amir Abdolkarimi, Sajjad Alizadeh, Maryam Karimian

**Affiliations:** 1 Department of Physiology, Tehran University of Medical Sciences, Tehran, Iran; 2 Department of Physiology, Arak University of Medical Sciences, Arak, Iran; 3 Clinical Biochemistry Research Center, Shahre Kurd University of Medical Sciences, Shahre Kurd, Iran; 4 Faculty of Medicine, Ilam University of Medical Sciences, Ilam, Iran; 5 Faculty of Medicine, Kermanshah University of Medical Sciences, Kermanshah, Iran

**Keywords:** Breathing pattern, Dynamic compliance, Respiratory impedance, Unilateral nasal stimulation

## Abstract

***Objective(s):*** Reflexes that rose from mechanoreceptors in nasal cavities have extensive neuro-regulatory effects on respiratory system. Because of side specific geometry and dual innervations of the nasal mucosa, we investigated the consequences of unilateral nasal stimulations on respiratory mechanics and breathing patterns.

***Materials and Methods:*** Unilateral nasal air-puff stimulation (30 min) in the presence of propranolol (25 mg/kg) and atropine (5 mg/kg) were applied on tracheotomized spontaneously breathing rats. Breathing rate and pattern monitored. Peak inspiratory pressure (PIP) and flow (PIF) were exploited for calculation of resistance, dynamic compliance (C_dyn_), and estimation of respiratory system impedance (Z_rs_).

***Results:*** During right-side stimulation, in propranolol (P<0.05) and atropine groups (P<0.01) PIP significantly decreased in comparison to the control group. Alternatively, it significantly increased in left-side and propranolol-left groups (P<0.05) than control group. Mean C_dyn_ following left-side stimulation and propranolol, revealed significant decrements (P<0.05) than control group. In the case of atropine-right and atropine-left groups, mean C_dyn_ had significantly decreased in comparison with atropine alone (P<0.05). Airway resistance (R) did not reveal significant difference during nasal stimulations whereas least square approximation revealed a significant side-specific frequency dependent deviation of imaginary part of impedance (X). An inverse correlation was determined for C_dyn_ versus frequency following right side (R=-0.76) and left side (R=-0.53) stimulations.

***Conclusion:*** For the reason that lower airways mechanics changed in a way independent from smooth muscle, it may be concluded from our data that unilateral nasal stimulations exert their different controls through higher regulatory centers.

## Introduction

Despite of widespread cosmetic surgical operations of the nasal area, little concern is about to the physiological importance of the nasal cavities in the respiratory system, other than to warm up and moisturize the fresh air being supplied to the alveoli. Comprehensive search revealed no reliable documentation of possible post-operational side effects in the developing countries despite their high rate of cosmetic surgeries specific to the nasal area. Whilst from the gross respiratory system physics to the nuclear receptors of non-respiratory–associated chemicals inside the cell (1), all participate in shaping the final pattern of a breath. Future developments in cosmetic surgeries and intranasal drug delivery approaches (2) may be inclusive for complex structure and function of the upper airways.   

Numerous simulations have shown that air flow properties through the nasal cavity are highly sensitive to the geometry of the passageway which could be straightaway affected even following minor repairs (3, 4). Altered fluid properties could lead to stress and refuse of energy dissipation inside, which in turn results in the disturbance of dynamic equilibrium and the firing rate of mechanoreceptors devoted to the nasal mucosa (5). Afterwards, propagated discharges travel in either side of nasal septum to the central sensory and regulatory nuclei of the brainstem, such as solitary tract nucleus (NTS) and nucleus ambiguous in which several circuits are modifying respiratory reflexes (6). Activation of pump cells (*P-*cells) in NTS may be modulated via nasal receptors which thereby modify the input to the ventrolateral medulla involved in control of respiratory motor pattern (7). Transient inhibition of respiratory motor centers are associated with activation of bulbar expiratory units, therefore the activation of nasal afferents on medullary respiratory neurons could be well demonstrated on responses involved in ventilation and airway patency (8).

Enhanced elucidation of reflex phenomena involves uncovering the relationships between key mechanical determinants, such as changes of rate (*f*), tidal ventilation (V_T_), trans-pulmonary pressure (P_tp_) and complex indicators consist of airway resistance (R), dynamic compliance (C_dyn_) and respiratory system impedance (Z_rs_) following nasal stimulation. Former investigations revealed that application of electrical stimulation (9) and alternative air-puff (8), and nasal dry and cold-dry air stimulation (10), separately could affect at least one of the aforementioned variables in dogs, guinea pigs, cats, and healthy individuals. Furthermore, side-specific dominancy following the so called *Pranayamic *nasal breathing has been stated in recent investigation about blood pressure, pulmonary function, relaxation, cognition and concentration in humans (11-14).

As it happens, the relationship between the upper and lower airways in the pathogenesis of respiratory abnormalities has been proposed for mechanism(s) that remain inadequately understood. Considerable information has been intuitively grasped from asthmatic individuals with nasal abnormalities, in which high levels of oxidant load and inflammatory mediators such as nitric oxide (NO) concentration in the airways were revealed (15,-18). This information naturally raises a question as to which respiratory structure or function could be involved in this phenomenon. This condition could not be elucidated however, unless detailed mechanical properties of the respiratory system were understood. There is a trend favoring the benefits of spectral impedance measurements in the study of respiratory mechanics, therefore in order to increase our knowledge about changes of resistance (R) and reactance (X) a synergistic integration of flow and pressure monitoring for impedance measurement, we performed the unilateral nasal air-puff stimulation in rats. Consequently the aim of this study was to find out a detailed evaluation of the effects of side-specific nasal stimulations on dynamic compliance, respiratory system impedance and breathing patterns.

## Materials and Methods

This study was performed in accordance with the Tehran University of Medical Sciences ethical instructions in animal research and experiments.

*Design*

Forty five *NMRI* rats of either sex between 8-12 weeks old, weighing 150–230 g, were included. All animals were housed under standard conditions until the time of experiments. They randomly divided into control and sham-operated and two main unilateral nasal stimulation groups of right side (RS) and left side (LS) of five rats per each and experiments started in a daily time range from 10 AM to 2 PM.  In sham-operated group, an identical surgical procedure was performed and the catheter was put on the nostril without stimulation (see below). Single dose of atropine (5mg/kg) and propranolol (25mg/kg) injected intraperitoneally 10 min prior to stimulation in relevant groups and co-administered in a separated group.

*Nasal air-puff stimulation*

Animals initially were anesthetized with ketamine hydrochloride (75 mg/kg) and xylasine (5 mg/kg) given intraperitoneally (IP). Adequate anesthesia was assured with corneal and pedal reflexes. The trachea was exposed through a midline incision on a surgical warming pad and cannulated low in the neck and the esophagus was ligated. Laryngeal nerves were sectioned bilaterally with a glass hook. Nasal air-puff stimulation (23°C, 5 L/min, 65/min) was delivered continuously with a conventional respirator (Palmer R2750 England) for 30 min (room air) ipsilaterally through a polyethylene catheter (ID: 1.3 mm), 5 mm beyond the nostril opening. Contralateral nostril was sutured and the duration of air puffs was kept constant at 0.2s and augmentation of the intrinsic activity of larynx as the effectiveness of stimulation was confirmed. 

*Data collection*

Electrocardiography (ECG), blood pressure (data not shown), airway flow and pressure signals were recorded by a narcobiosys model physiograph for the full duration of the experiment, which lasted approximately 30 min, and air-puff stimulation was continuously applied to the nasal cavity. Dedicated non-heated piezoresistive flow (Honeywell ±3SLPM transmitter) and pressure transducers (Validyne MP445 Differential) for respiratory measurements were used during quiet breathing. Tracheal pressure was recorded from the side arm of the airway opening. 

The very short data sampling intervals and the high power of time resolution applied during the calculations which almost assured a quasi-linearity of dynamic compliance. 

*Data analysis*

*Lung mechanics*

For measuring C_dyn_, first derivative of tidal volume was exploited by reading its interpolated values from the inspiratory phase divided by PIP with random selection criteria of 8–10 breaths.

For elimination of the influence on the respiration rate, averaged values from the inspiratory phases accordingly were divided by the frequency of respiration of the animal.  Respiratory system impedance (was calculated from the interpolated values of R and X as a function of frequency (f). 

(ω) = R (ω) + jX (ω)

Where            *and*           ω= 2π*f*

From the young module implementation, we expressed X as elastance (E) as a function of*f*. Therefor;

Estimation of Z_rs_ was obtained from the flow and pressure signals by a least-squares algorithm described elsewhere (19, 20). Finally, for a full inferential assessment hidden inside the measured parameters, we used equation of motion for airway pressure (against changes in resistive and elastic properties of the respiratory system. 

Where stands for flow rate. Impedance data were computed for the full duration of the experiment, creating a continuous tracing of R_rs_, X_rs_. These values were averaged over the periods of inspiratory phase providing a single impedance data point for each.

*Statistical analysis*

Scattered data were expressed as mean ± SD. The relationship between respiratory rate (*f*) and C_dyn_ was evaluated by linear regression analysis, and the correlation coefficient (r) was calculated. We performed the linear regression analysis intra-individually and as there were different experimental interventions for each stimulation group; we performed one-way ANOVA and Tukey posttest. Statistical significance was accepted at the *p <* 0.05 level. 

A Wilcoxon signed rank test was used to determine any significant difference between collected data at different time of experiments for normalized pressure and flow recordings.

## Results

Changes in amplitude of breaths, and frequency of respiration

**Figure 1:**
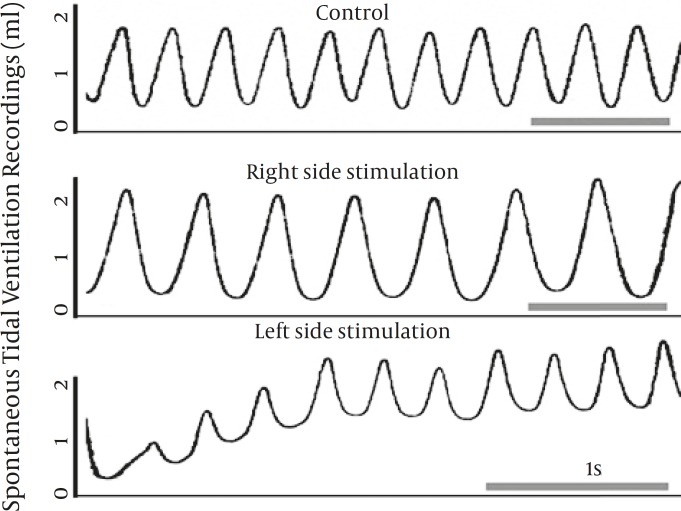
Representative tidal airflow tracing of three spontaneously breathing animals of control and stimulation groups. The bar indicates the time equal to 1s. Note that following LS, multiple sighs occurred and increased functional residual capacity (FRC) in several succeeding breaths after the sigh is indicated

Figure 1 reveals the representative flow tracing of three experiments, respectively. As it is shown, there are considerable changes in the frequency and tidal ventilation following RS and LS. In lower tracing repetitive sighs were followed by changes in tidal flow and functional residual capacity (FRC) from the base line.

Table 1 shows the mean values of PIP, tidal flow (F_T_), frequency of breathing (*f*) and mass-specific ventilation (V_E_) of spontaneously breathing rats among control and during RS, LS, and atropine and propranolol treatments. We didn’t monitor the FE_Co2_ in our experiments, but as it is derived from the calculation of *V*_E_, there are significant differences in the V_E_ of LS group, in spite of considerable changes in *F*_T _and *f*.

Effect of the IP injection of atropine and propranolol

Administration of atropine significantly decreased breathing frequency in comparison to the control group (*P<*0.05) (table 1), whereas FT, did not reveal a significant difference. Propranolol by itself increased f with a significant difference than the control group (*P<*0.01). Student t-test revealed a significant difference in PIP between atropine and propranolol. Finally one-way ANOVA showed significant changes of PIP value between Atr and RS (*P<*0.05) and LS (*P<*0.01), respectively. Further analyses did not show significant differences for C_dyn_, and total Rrs. ANOVA showed significant differences of V_E_ among LS, Atr+LS and Prop+LS, respectively (*P<*0.05) (Table 1). With respect to RS and LS, atropine significantly decreased PIP with no significant changes in FT, f and V_E_ (*P<*0.05 & *P*<0.01, respectively).

**Table 1:** Descriptive values of respiratory parameters in stimulated and non-stimulated groups with different treatments during spontaneous breathing

	PIP		*F*_T_	*f *	VE
	cmH_2_O	ml s^-1^	Breaths min^-1^	ml g^-1 ^min^-1^	
Control	1.93±0.1		4.06±0.91	102.6±18.24	2.82±0.23
RS	2.25±0.07		3.51±0.62	116.1±8.1*	2.54±0.54
LS	3.16±0.15 *		2.86±0.30 *	128.4.±7.6 *	2.29±0.28 *
Prop	2.62±1.71		2.94±1.19	186.8±9.9 **	3.41±0.73
Atr	1.73±0.02 # ††		3.88±1.1	124.7±10.3	3.02±1.2
Atr +RS	2.05±0.16		2.91±1.22	147.3±8.24	2.67±1.54
Atr + LS	2.24±1.85		2.85±0.80	113.6±19.1	1.75±1.82* †
Prop + RS	2.25±0.07		3.51±0.62	141.6±21.5*	3.1±0.54
Prop + LS	3.16±0.15 *		2.92±1.30	112.6±28.3	1.77±0.48 *

Values are mean±SEM. PIP: peak inspiratory pressure, FT: tidal Flow, f: frequency of respiration and VE: mass-specific ventilation. RS; right stimulation, LS; left stimulation, Prop; Propranolol, Atr; Atropine.*=P<0.05 to the control, **=P<0.01 to the control, #=P<0.05 to the RS,†=P<0.05 to the LS, ††=P<0.01 to the LS. n=5

*Changes in airway resistance, and inspiratory dynamic compliance*

**Figure 2:**
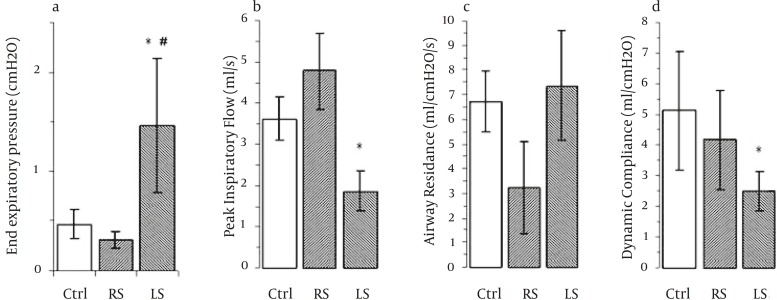
End expiratory pressure, inspiratory flow, resistance and dynamic compliance of the three different groups (n=6). Ctrl; control, RS; right side stimulation, LS; left side stimulation. *= P <0.05 to the control, #= P <0.05 to RS

Left side stimulation revealed significant difference in end expiratory pressure (EXP) as compared to control and RS (*P<*0.05) (Figure 2). Changes in pressure are considerably affected in EXP and PIP (Table 1 and Figure 2a). A non-statistically significant trend was apparent in R following right side stimulation, (Figure 2c). Finally dynamic compliance () measured from inspiratory flow and pressure was significantly decreased after LS (*P*<0.05) (Figure 2d).

**Figure 3:**
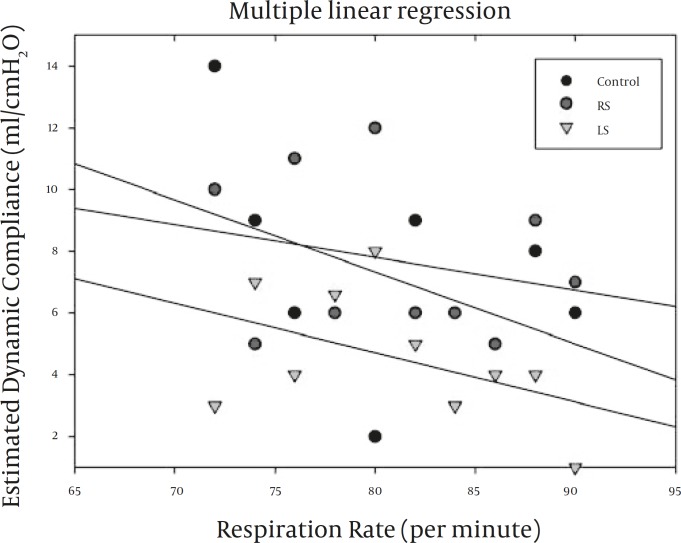
Multiple scattered plot of approximated dynamic lung compliance as a function of different respiratory frequencies in control and following unilateral nasal air-puff stimulation. RS: right side stimulation, LS: left side stimulation

Figure 3 depicts multiple scattered plot of estimated dynamic compliance of control and unilateral nasal air-puff stimulations. A full inferential concept is frequency dependence of the C_dyn_ to increased frequency even in the control group. Unilateral nasal stimulation non-uniformly changed the slope and steepness of the compliance trend line.

*Modeled resistance and reactance in the impedance spectrum*

**Figure 4:**
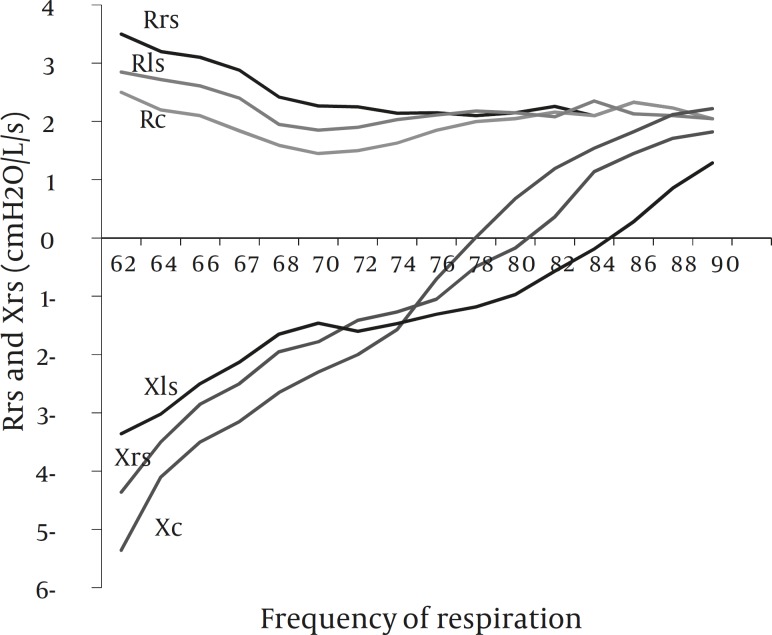
Changes in modeled resistance (R) and reactance (X) of the respiratory system due to an increase in frequency of breathing. Intrinsic mechanical properties estimated prior to the approximation used to compute the modeled R and X. Data shown here are mean value of the R and X placed in the model described above without application of the fitting improvement criteria

Figure 4 shows the modeled curves of the impedance spectrum consisting of resistance (R) and reactance (X) plotted in the frequency domain. Complex parameters were calculated from the intrinsic mechanical properties of the respiratory system. Although no improvement applied to the data, it is apparent that reactive component has a considerable variation among these groups. Crosses of the reactive curves to the abscissa represent the resonant frequency (f_res_) which denote the frequencies at which the equality of capacitive and inertive property of the reactance are met.

## Discussion

Air puff stimulation enabled us to study respiratory parameters occurring in parallel during quiet breathing. As shown in Figure 1, tidal flow show considerable changes during quiet breathing. There are significant changes of frequency following nasal stimulation. As it is inferred from the middle tracing, there is a moderate increase in the steepness of the flow recording following RS. On the other hand, lower tracing shows respiration pattern after LS which is substantially altered from normal rhythm. Attenuation of the slope of inspiratory phase and late-expiratory upward deflections indicate the possible air-trapping (21). Therefore increased FRC following successive cycles is a strong determinant of development of dynamic hyperinflation. Principal among our result is that the net effect of each side of stimulation is significantly different in case of FT, C_dyn_ and X_rs_. Our only option is to infer what is going on in the airways such that it is different for each side. 

Estimated C_dyn_plotted against the f with averages extrapolated from the calculated quantities revealed significant deviation, and emphasizes attenuation of C_dyn_ due to increased f as mentioned by other studies (21, 22). In Figure 3, both RS and LS inclined to reduce C_dyn_ with increasing f. Decreased C_dyn_ could be the result of increased FRC and the stretching of airways due to hyperinflation and air trapping especially following LS. A comparison between Figure 3 and 4, indicate the f ≈75Hz as a critical frequency at which C_dyn_ and X_rs_ are switched behind between RS and LS.

The relationship between lung compliance and f has been the subject of several former studies (22-24) in which it is obvious that the frequency-dependence of compliance occurs in situations of obstruction (25). According to the theory of Otis *et al*. (1956), frequency-dependence of C_dyn_ implies the beginning of compartmentation with two different time constants that point out the distribution of inspired gas by single and double exponential functions. 

Former studies could not give a complete description of mechanical changes of the respiratory system in response to nasal stimulations as the authors did not provide a detailed explanation as to which lung properties are involved. Thus an altered ventilatory pattern itself may affect the lung mechanics so that active effects of nasal stimulation are biased (26-28). While the airway resistance (Raw) determined in the present study is consistent with those reported previously, the current X_rs_ appear to be somewhat different. This property is highly dependent on fluid velocity and physical driving forces, which extends as far down as the alveolar ducts.

Unilateral nasal stimulation which leads to increase in f all had a decrease in C_dyn_. At lower f (<77/min), RS tends to increase C_dyn_ compared to control, however because of greater slope, it reveals decreasing values afterwards. The continuity of the lines make it noteworthy as it would soon cross the LS line in higher fs, which reveals the complex effects on viscoelastic properties of the respiratory system. These properties may be mediated independently from the activation of smooth muscles, which means that our experiment with administration of atropine didn’t reveal considerable changes in FT or C_dyn_. Furthermore, changes in FT reside beyond these changes less than f, which provides additional support for this idea. 

Our results also support the assumption of non-uniformly distributed ventilatory lung volume ratio. It is challenging to explain, however, why there should be a correlation between this stratified inhomogeneity and frequency-dependence of compliance. Although in accordance to Woolcock *et al*. (29), our data suggests nearly more than 20% decrease in C_dyn_ at f>70/min, which might be considered as peripheral airway narrowing. Non-uniformly distributed ventilation and that possible recruitment of the remaining compartments may be responsible for undetectable alterations of total lung resistance in those studies. 

Spectral analysis of Z_rs_ showed prominent changes of reactance (X_rs_) by a frequency dependent manner. As it is shown in the Figure 4, X_rs_ is highly deviated to the right following LS, to the extent that zero-line crossing is away from control. This is asignificant characteristic of X_rs_ because this point is equivalent to the resonance frequency (f_res_) which balances capacitive and inertive ones, so deviation from the control f_res_ states an alteration in the dynamic constraints which implies the possible changes in smooth muscle tone and thickness of airways. 

## Conclusion

Our data propose that C_dyn_ is changed following unilateral nasal stimulation in a frequency-dependent manner which is significantly different among RS and LS. Because of reactive and capacitive changes of the lower airways in a way independent from smooth muscle, it may be concluded that nasal stimulation exert its effects through higher control centers.
